# Circulating levels of mitochondrial uncoupling protein 2, but not prohibitin, are lower in humans with type 2 diabetes and correlate with brachial artery flow-mediated dilation

**DOI:** 10.1186/s12933-019-0956-4

**Published:** 2019-11-09

**Authors:** Mamatha Kakarla, Venkata K. Puppala, Sudhi Tyagi, Amberly Anger, Kathryn Repp, Jingli Wang, Rong Ying, Michael E. Widlansky

**Affiliations:** 1 Department of Medicine, Division of Cardiovascular Medicine, Medical College of Wisconsin, Hub for Collaborative Medicine, 5th Floor A5743, 8701 W. Watertown Plank Road, Milwaukee, WI 53226 USA; 2 Department of Pharmacology, Division of Cardiovascular Medicine, Medical College of Wisconsin, Milwaukee, WI USA

**Keywords:** Mitochondria, Endothelium, Mitochondrial membrane potential, UCP2, PHB

## Abstract

**Background:**

Excessive reactive oxygen species from endothelial mitochondria in type 2 diabetes individuals (T2DM) may occur through multiple related mechanisms, including production of mitochondrial reactive oxygen species (mtROS), inner mitochondrial membrane (Δψ_m_) hyperpolarization, changes in mitochondrial mass and membrane composition, and fission of the mitochondrial networks. Inner mitochondrial membrane proteins uncoupling protein-2 (UCP2) and prohibitin (PHB) can favorably impact mtROS and mitochondrial membrane potential (Δψ_m_). Circulating levels of UCP2 and PHB could potentially serve as biomarker surrogates for vascular health in patients with and without T2DM.

**Methods:**

Plasma samples and data from a total of 107 individuals with (N = 52) and without T2DM (N = 55) were included in this study. Brachial artery flow mediated dilation (FMD) was measured by ultrasound. ELISA was performed to measure serum concentrations of PHB1 and UCP2. Mitochondrial membrane potential was measured from isolated leukocytes using JC-1 dye.

**Results:**

Serum UCP2 levels were significantly lower in T2DM subjects compared to control subjects (3.01 ± 0.34 vs. 4.11 ± 0.41 ng/mL, P = 0.04). There were no significant differences in levels of serum PHB. UCP2 levels significantly and positively correlated with FMDmm (r = 0.30, P = 0.03) in T2DM subjects only and remained significant after multivariable adjustment. Within T2DM subjects, serum PHB levels were significantly and negatively correlated with UCP2 levels (ρ = − 0.35, P = 0.03).

**Conclusion:**

Circulating UCP2 levels are lower in T2DM patients and correlate with endothelium-dependent vasodilation in conduit vessels. UCP2 could be biomarker surrogate for overall vascular health in patients with T2DM and merits additional investigation.

## Background

The prevalence of diabetes mellitus (DM) is increasing worldwide with an estimated 425 million people with DM in 2017 [1]. Diabetes mellitus type 2 (T2DM) is a chronic metabolic disease associated with dysfunction and failure of multiple organ systems (e.g. heart, renal, visual, and vascular) [2]. The majority of morbidity and mortality in patients with T2DM is secondary to large and small vessel vascular disease. Clinical manifestations are preceded by development of vascular endothelial dysfunction. While multiple factors contribute to endothelial dysfunction in T2DM, increased production of reactive oxygen species from mitochondria (mtROS) significantly contribute to vascular endothelial dysfunction [3–6].

Excessive endothelial mtROS production in T2DM occurs through multiple related mechanisms, including inner mitochondrial membrane hyperpolarization, changes in mitochondrial mass and membrane composition, and fission of the mitochondrial networks [7–12]. Partial mitochondrial membrane depolarization of arterioles improve endothelium-dependent vasodilation and NO bioavailability in arterioles from patients with T2DM or arterioles from health individuals exposed acutely to abnormal glucose levels [7, 8, 13]. These data suggest proteins involved with regulating mitochondrial membrane potential could potentially serve as biomarker surrogates for vascular health in T2DM [7, 14]. This concept is further supported by data suggesting mitochondrial-derived proteins and nucleotides may also serve play paracrine and endocrine-like signaling roles regulating vascular health [15, 16].

Overexpression of two proteins that regulate mitochondrial membrane potential, uncoupling protein-2 (UCP2) and prohibitin (PHB), reduce mitochondrial membrane potential (Δψ_m_) [17–19]. Prohibitin proteins assemble super-complexes of electron transport chain proteins allowing for efficient flux of electrons and reducing levels of mtROS production [20]. UCP2 overexpression also reduces mtROS production, reduces atherosclerosis formation in animal models, improves coronary artery endothelium-dependent vasodilation, and is associated with reduce risk of myocardial infarction in humans [21–23]. These data suggest UCP2 and PHB could be potentially be useful biomarker surrogates for human vascular endothelial health but their utility in this scenario remains unknown. The main objective of this pilot study is to determine whether circulating levels of UCP2 and PHB correlate with brachial artery flow-mediated dilation (FMD%), a well-accepted measure of in vivo human endothelial function and surrogate marker of cardiovascular risk [24, 25], in individuals with and without type 2 diabetes.

## Methods

### Subjects

107 individuals with (N = 52) and without type 2 diabetes (N = 55) consecutive individuals, ages 35–70 without prevalent cardiovascular disease, were recruited based on inclusion and exclusion criteria as previously described, agreed to banking of their study information and blood and tissue samples under a process approved by the Medical College of Wisconsin’s Institutional Research Board, and had available serum samples for analyses [7], The diagnosis of T2DM was made by the subject’s primary provider based on standard American Diabetes Association criteria at the time of enrollment. Samples and data from sixty-four subjects from prior published studies were included in this new analysis [7]. Individuals without T2DM were healthy individuals without traditional cardiovascular risk factors (non-smokers, no hypertension, no elevated lipids). Any potential subject with an elevate baseline plasma creatinine (> 1.5 mg/dL in men and > 1.4 mg/dL in women), a history of cardiovascular disease (including clinical history of stroke, peripheral vascular disease, myocardial infarction, or documented ≥ 50% stenosis of ≥ 1 major epicardial coronary artery), a diagnosis of type 1 diabetes, a history of a major chronic illness, or a history of cigarette smoking within 1 year of study enrollment were excluded. All subjects had their height and weight measured and their heart rates and blood pressures measured in triplicate. Plasma and serum samples obtained from these subjects and stored at -80C. Mononuclear cells were isolated for measurement of Δψ_m_ as previously described [9].

### Measurement of brachial artery reactivity

Brachial artery reactivity is a well-validated measure of systemic endothelial function in humans that is known to be predictive of future adverse cardiovascular events and modifiable by therapies know to impact cardiovascular risk [24–26]. Brachial artery endothelial function was measured using high resolution ultrasound [Logiq 500Pro (GE) or MicroMaxx (Sonosite)] as previously described [27–31]. Briefly, following an overnight fast, subjects arrived at MCW’s Translation Research Unit and were allowed to rest in a temperature controlled, quiet room for 15 min prior measurements. A blood pressure cuff was placed below the cubital fossa of the dominant arm that was supinated and abducted approximately 80° from the subject’s torso. The brachial artery was imaged 1–3 cm cephalad from the cubital fossa. Following acquisition of the resting brachial artery diameter and flow velocities, the blood pressure cuff was inflated to the greater of 50 mmHg above systolic pressure or 200 mmHg and flow occlusion verified. After 5 min of cuff occlusion, the blood pressure cuff was deflated and the brachial artery diameters and hyperemic flow velocities were measured as previously described [29]. All analyses were performed by trained technicians using established software (Brachial Analyzer, Medical Imaging Applications LLC) and our intra- and inter-observer reproducibility data has been previously reported [27]. Endothelial function is reported as absolute flow-mediated dilation from baseline diameter (FMDmm).

### Measurement of mitochondrial membrane potential

Measurement of mitochondrial membrane potential (Δψ_m_) was performed in mononuclear cells isolated from all subjects using a cationic dye, 5,5′,6,6′-tetrachloro-1,1′3,3′-tetraethylbenzamidazol-carboncyanine (JC-1; Invitrogen, Carlsbad, Calif) and applying the Nernst equation previously described [7, 9]. Reproducibility measurements for Δψ_m_ are as previously reported and all measurements were made in triplicate and averaged to derive the point estimate of Δψ_m_ for each subject. The membrane potential was calculated by using the Nernst equation: Δψ_m_ = − 60 × log[(JC-1 Red Fluorescence Intensity/JC-1 Green Fluorescence Intensity) × 0.0417]− 30 mV. The 0.0417 is used to approximate the percent cell volume of mitochondria in endothelial cells [32], and 30 mV are subtracted to account for the plasma membrane potential [33].

### Measurement of glucose, prohibitin, and UCP2

The plasma glucose was run by Wisconsin Diagnostics Laboratory using a hexokinase assay. Sandwich ELISA (Enzyme linked immunosorbent assay) kits were used to determine the quantitative levels of PHB (LS-F6361, LifeSpan Biosciences, Seattle, WA and UCP2 (LS-F21119, LifeSpan Biosciences, Seattle, WA) in human serum samples. All the reagents and standards were prepared following the manufacturers’ instructions. In each plate, standards and samples were run in duplicates. To test for variation between the plates, four samples (two T2DM and two healthy control) were run as inter-assay controls. The inter-assay correlations between samples run on different plates for PHB were 0.85 in T2DM subjects and 0.90 in healthy controls. Similarly, for UCP2, the inter-assay correlation values were 0.82 in T2DM subjects and 0.77 in healthy control subjects. The loaded plates were read at 450 nm in a SpectraMax M5e Microplate Reader (Molecular Devices, Sunnyvale, CA) and optical density (O.D.) was measured. The O.D. readings for each standard and sample was averaged between the duplicates. For the samples that were used as inter assay controls, the readings were averaged across the two plates.

### Statistical analyses

Statistical analyses were performed using R (Version 3.4.0) [34] and SPSS 24.0 (IBM). Missing data values for prohibitin (N = 8), UCP2 (N = 2), SBP (N = 2), DBP (N = 2), HR (N = 2) were imputed using baseline characteristics (age, sex, ethnicity, history of diabetes, body mass index, systolic and diastolic blood pressure, resting heart rate, fasting glucose, cholesterol levels) with UCP2 and PHB levels and pooling five imputations. The imputed variables are reported as mean ± SEM while all other continuous variables are reported as mean ± SD. Analyses were done on the imputed dataset. Baseline characteristics were compared between T2DM and non-T2DM controls using unpaired t-tests, Mann–Whitney U tests, Chi square tests, or Fisher’s Exact Test as appropriate. Measurements of endothelial function, Δψ_m_, prohibitin, and UCP2 were similarly compared between groups. Pearson r correlation coefficients were calculated to determine if there were associations between measures of endothelial function, Δψ_m_, and risk factors with circulating levels of prohibitin, while Spearman’s rho were used to determine associations with UCP2 (levels not normally distributed). Age- and sex-adjust linear regression models were created to determine the independent predictive value of the mitochondrial proteins and Δψ_m_ for vascular function measurements that showed significant correlations. Additional models including fasting glucose, BMI ≥ 30 kg/m^2^, and systolic blood pressure > 130 mmHg were performed to determine the independent predictive value of mitochondrial proteins and Δψ_m_ that were predictive in the age- and sex-adjusted models. P values < 0.05 were considered significant.

## Results

### Subjects

The baseline characteristics of the study population is included in Table 1. When comparing T2DM subjects to controls, T2DM subjects had a significantly higher mean body mass index, age, systolic blood pressure, resting heart rate, fasting glucose, and triglyceride levels. Total and LDL cholesterol levels were lower in T2DM subjects likely due to great use of HMG-CoA reductase medications in T2DM patients 51% were on HMG-CoA inhibitors. Full data on medication use in the T2DM subject group is included in Table 1. There were no significant differences in circulating UCP2 levels for those on HMG-CoA reductase inhibitors (3.2 ± 2.3 ng/mL) compared to those not on a medication in this class (2.8 ± 2.3 ng/mL, P = 0.50). Similarly there was no differences in circulating PHB levels between those on HMG-CoA reductase inhibitors (12.9 ± 4.3 ng/mL) compared to those not on a medication from this class (14.6 ± 5.8 ng/mL, P = 0.24). In addition, there was no significant difference in UCP2 (3.03 ± 2.25 vs. 2.92 ± 2.90 ng/mL, P = 0.90) or PHB levels (13.6 ± 4.4 vs. 14.0 ± 7.5 ng/mL, P = 0.81) for T2DM subjects on metformin versus those not on metformin. No significant differences in UCP2 (3.68 ± 2.25 vs. 2.72 ± 2.15 ng/mL P = 0.19) or PHB (12.8 ± 5.6 vs. 14.1 ± 4.9 ng/mL P = 0.19) levels were seen comparing T2DM subjects on sulfonylureas versus those not on sulfonylureas. No control subjects were on any vasoactive or cardioprotective medications.

**Table 1:** Baseline characteristics

	Total study population (N = 107)	Healthy controls (N = 52)	Type 2 DM (N = 55)	P-value T2DM vs. control
Age (years)	51 ± 10	46 ± 9	56 ± 8	< 0.001
Sex (% women)	55.5	58.2	55.8	0.80
Race				0.58
Caucasian (%)	82.2	81.8	82.7	
African American (%)	14.0	11.7	15.4	
Other (%)	0.9	1.8	1.9	
Not reported (%)	2.8	3.6	1	
Hispanic (%)	1.9	3.7	0	0.50
Body mass index (kg/m^2^)	31.9 ± 8.0	28.3 ± 6.2	35.7 ± 8.0	< 0.001
Systolic blood pressure (mmHg)	126 ± 17	120 ± 2	133 ± 12	< 0.001
Diastolic blood pressure (mmHg)	72 ± 10	72 ± 1	72 ± 1	0.85
Heart rate (beats/min)^a^	67 ± 11	65 ± 1	69 ± 1	0.018
Total cholesterol (mg/dL)	179 ± 39	190 ± 40	167 ± 35	0.002
LDL cholesterol (mg/dL)	102 ± 35	115 ± 34	88 ± 33	< 0.001
HDL cholesterol (mg/dL)	53 ± 15	55 ± 16	50 ± 14	0.10
Triglycerides (mg/dL)	127 ± 79	101 ± 58	154 ± 90	< 0.001
Glucose (mg/dL)	104 ± 38	82 ± 10	127 ± 44	< 0.001
Aspirin (% use)	21.5	1.9	40	< 0.01
Ace inhibitors/ARBs (% use)	32.7	0	63.6	< 0.01
HMG CoA reductase inhibitors (% use)	26.1	0	50.9	< 0.01
β-blockers (% use)	9.3	0	18.2	< 0.01
Calcium channel blockers (% use)	9.3	0	18.2	< 0.01
Metformin (% use)	39.3	0	80.7	< 0.01
Sulfonylureas (% use)	14.0	0	28.8	< 0.01
Thiazolidinediones (% use)	1.9	0	3.9	< 0.01
DPP4 inhibitors (% use)	0.9	0	1.9	NA
SGLT2 inhibitors (% use)	0	0	0	NA
GLP-1 agonists (% use)	0	0	0	NA
Insulin (% use)	0.9	0	1.9	NA
Prohibitin (ng/mL)	13.6 ± 0.6	13.4 ± 0.8	13.9 ± 0.7	0.66
UCP2 (ng/mL)	3.58 ± 0.27	4.11 ± 0.41	3.01 ± 0.34	0.045
Δψ_m_ (mV)	− 181 ± 10	− 177 ± 11	− 185 ± 9	< 0.001

^a^N = 39 healthy controls and N = 49 T2DM subjects

### Comparing FMDmm and nitroglycerin-mediated vasodilation in T2DM versus control subjects

The brachial flow-mediated dilation (FMDmm) was significantly lower in diabetic subjects compared with non-diabetic subjects (0.12 ± 0.06 vs. 0.26 ± 0.06 mm; P < 0.0001). No differences were seen in resting and peak flow velocities between T2DM subjects and controls. In the subjects that agreed to nitroglycerin use (N = 39 controls, N = 49 T2DM subjects), nitroglycerin-mediated dilation (NMDmm) was also lower in T2DM subjects (0.68 ± 0.25 vs. 0.85 ± 0.25 mm, P < 0.001) (Table 2).

**Table 2:** Brachial artery vascular measurements

	Total study population (N = 107)	Healthy controls (N = 52)	Type 2 DM (N = 55)	P-value T2DM vs. control
Resting diameter (mm)	3.72 ± 0.72	3.66 ± 0.75	3.81 ± 0.69	0.29
Resting flow velocity (m/s)	0.54 ± 0.14	0.53 ± 0.13	0.56 ± 0.15	0.32
Hyperemic flow velocity (m/s)	0.99 ± 0.26	1.01 ± 0.25	0.97 ± 0.26	0.45
Absolute flow mediated dilation (mm)	0.20 ± 0.09	0.26 ± 0.06	0.12 ± 0.06	< 0.001
Nitroglycerin Mediated Dilation (mm)^a^	0.75 ± 0.25	0.85 ± 0.25	0.68 ± 0.22	< 0.001

^a^N = 39 healthy controls and N = 49 T2DM subjects

### Comparisons and associations between mitochondrial measurements

**Fig. 1:**
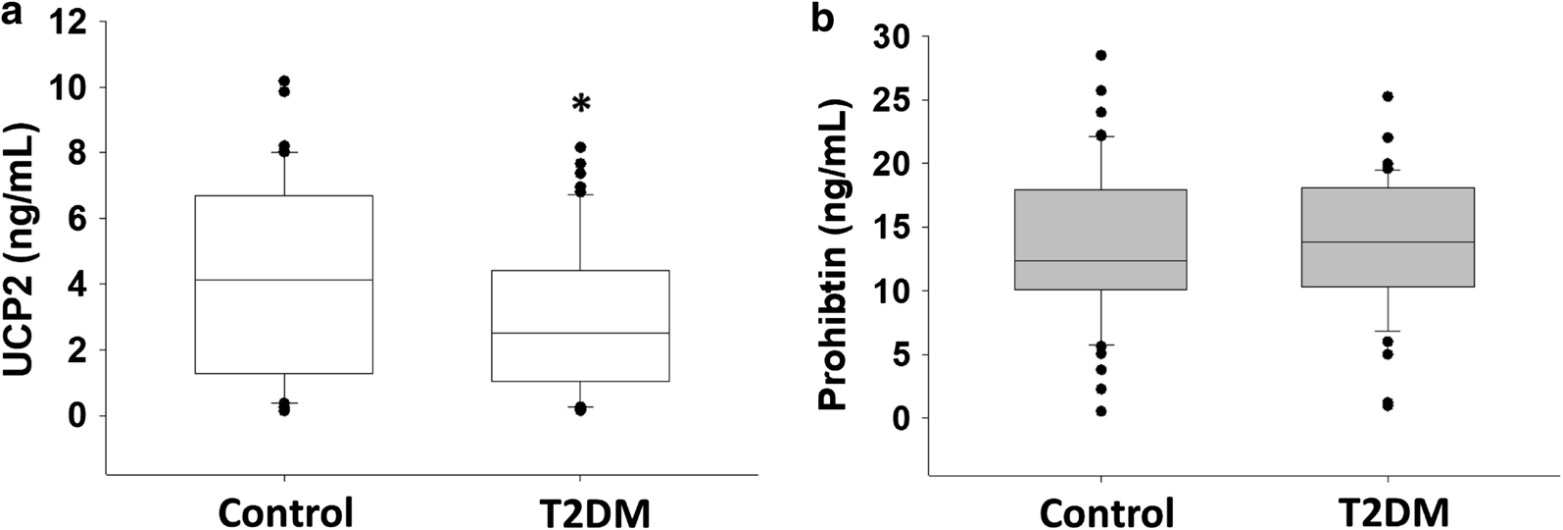
**a**, **b** Comparative serum levels of mitochondrial proteins in control and T2DM subjects. UCP2 levels were significantly higher in controls compared to T2DM subjects (p = 0.0418). **a** No significant differences in Prohibitin (p = 0.66). **b** Levels were seen between control and T2DM subjects

Serum UCP2 levels were significantly lower in T2DM subjects compared to controls (3.01 ± 0.34 vs. 4.11 ± 0.41 ng/mL P = 0.04, Fig. 1a). There were no significant differences in levels of serum PHB (13.9 ± 0.7 ng/mL for T2DM patients vs. 13.4 ± 0.8 ng/mL, P = 0.66) between T2DM and control subjects (Fig. 1b). Mononuclear cell inner mitochondrial membrane potential (Δψ_m_) was significantly more polarized in subjects with T2DM compared to controls (− 185 ± 8.7 vs. − 177 ± 11 mV, P < 0.001).

Within T2DM subjects, serum PHB levels were significantly and negatively correlated with UCP2 levels in both univariate analysis (ρ = − 0.35, P = 0.031) and following age- and sex- adjustment (Β = − 0.17, P = 0.03). Neither UCP2 levels (r = 0.14, P = 0.61) nor PHB levels (ρ = − 0.09, P = 0.53) correlated with Δψ_m_ in univariate analyses and this did not change with age- and sex-adjustment (PHB: β = − 0.02, P = 0.95, UCP2: β = 0.76, P = 0.17). In healthy controls, there was a trend toward a negative correlation between PHB and UCP2 (ρ = − 0.27, P = 0.06) which was similar following adjustment for age and sex (β = − 0.14, P = 0.06). There was a positive correlation between UCP2 concentrations and Δψ_m_ (r = 0.31, P = 0.02) that remained following adjustment for age and sex (β = 1.1, P = 0.02) with no correlation between PHB and Δψ_m_ in either univariate (ρ = − 0.03, P = 0.82) or age and sex-adjusted models (β = − 0.15, P = 0.54).

### Univariate correlations between circulating mitochondrial peptide levels and Δψ_m_ with age, sex, and other cardiovascular risk factors

Univariate correlations between mitochondrial measures and age, sex, and traditional risk factors in T2DM patients are include in Table 3 and for healthy controls in Table 4. For T2DM subjects, positive correlations between UCP2 and BMI (r = 0.32, P = 0.04) and between Δψ_m_ and total cholesterol (r = 0.31, P = 0.02) were detected. In addition, total cholesterol negatively correlated with UCP2 levels (r = − 0.28, P = 0.046). In healthy controls, the only correlation between a mitochondrial biomarker and a cardiovascular risk factor was a negative correlation between PHB and HDL cholesterol (ρ = − 0.36, P = 0.008). No correlations were found between other cardiovascular risk factors (age, sex, systolic blood pressure, diastolic blood pressure, fasting blood glucose, HDL, LDL, or triglycerides).

**Table 3:** Univariate correlations of Δψ_m_, prohibitin, and UCP2 with clinical variables in T2DM subjects

	Δψ_m_	Prohibitin	UCP2
Age	r = − 0.08, P = 0.58	ρ = 0.12, P = 0.42	r = 0.05, P = 0.72
Sex	r = − 0.08, P = 0.58	ρ = 0.26, P = 0.07	r = 0.24, P = 0.09
SBP	r = 0.11, P = 0.46	ρ = 0.05, P = 0.71	r = − 0.05, P = 0.73
DBP	r = − 0.05, P = 0.74	ρ = 0.09, P = 0.54	r = − 0.07, P = 0.61
BMI	r = 0.12, P = 0.39	ρ = 0.06, P = 0.69	*r = 0.32, P = 0.04*
Fasting blood glucose	r = − 0.22, P = 0.11	ρ = − 0.10, P = 0.49	r = − 0.08, P = 0.60
Total cholesterol	*r = 0.29, P = 0.04*	ρ = 0.001, P = 0.99	*r = *− *0.28, P = 0.046*
LDL cholesterol	r = 0.18, P = 0.21	ρ = 0.04, P = 0.77	r = − 0.27, P = 0.07
HDL cholesterol	r = 0.15, P = 0.30	ρ = − 0.11, P = 0.47	r = − 0.21, P = 0.15
Triglycerides	r = 0.02, P = 0.87	ρ = 0.14, P = 0.33	r = 0.03, P = 0.89

Italic values indicate p < 0.05

**Table 4:** Univariate correlations of Δψ_m_, prohibitin, and UCP2 with clinical variables in healthy control subjects

	Δψ_m_	Prohibitin	UCP2
Age	r = − 0.04, P = 0.78	ρ = 0.06, P = 0.65	r = 0.004, P = 0.98
Sex	r = − 0.003, P = 0.98	ρ = 0.23, P = 0.10	r = 0.06, P = 0.66
SBP	r = − 0.06, P = 0.69	ρ = 0.01, P = 0.92	r = 0.17, P = 0.23
DBP	r = 0.13, P = 0.34	ρ = 0.13, P = 0.37	r = − 0.04, P = 0.75
BMI	r = − 0.09, P = 0.50	ρ = 0.08, P = 0.58	r = − 0.08, P = 0.55
Fasting blood glucose	r = 0.03, P = 0.85	ρ = − 0.16, P = 0.25	r = − 0.22, P = 0.10
Total cholesterol	r =− 0.08, P = 0.57	ρ = − 0.25, P = 0.07	r = 0.03, P = 0.85
LDL cholesterol	r = − 0.09, P = 0.54	ρ = − 0.17, P = 0.23	r = − 0.001, P = 0.99
HDL cholesterol	r = 0.11, P = 0.42	*ρ = *− *0.36, P = 0.008*	r = 0.02, P = 0.88
Triglycerides	r = − 0.20, P = 0.16	ρ = 0.09, P = 0.54	r = 0.07, P = 0.27

Italic values indicate p < 0.05

### Univariate correlations between circulating mitochondrial peptide levels, Δψ_m_, and vascular measures in T2DM subjects and healthy controls

Univariate correlations between mitochondrial measures and vascular measures are displayed in Table 5 (for T2DM patients) and Table 6 for healthy controls.

**Table 5:** Univariate correlations of Δψ_m_, prohibitin, and UCP2 with vascular measurements in T2DM subjects

	Δψ_m_	Prohibitin	UCP2
Resting diameter	r = − 0.01, P = 0.97	ρ = 0.22, P = 0.12	r = 0.14, P = 0.31
Resting flow velocity	r = − 0.02, P = 0.89	*ρ = − 0.28, P = 0.048*	r = 0.18, P = 0.62
Hyperemic flow velocity	r = − 0.002, P = 0.97	ρ = − 0.15, P = 0.29	r = 0.19, P = 0.18
Absolute flow mediated dilation	r = − 0.01, P = 0.95	ρ = − 0.052, P = 0.73	*r = − 0.30, P = 0.03*
Nitroglycerin mediated dilation	r = − 0.04, P = 0.80	ρ = 0.26, P = 0.09	r = − 0.30, P = 0.06

Italic values indicate p < 0.05

**Table 6:** Univariate correlations of Δψ_m_, prohibitin, and UCP2 with vascular measurements in healthy control subjects

	Δψ_m_	Prohibitin	UCP2
Resting diameter	r = − 0.02, P = 0.90	*ρ = 0.28, P = 0.049*	r = − 0.11, P = 0.43
Resting flow velocity	r = − 0.26, P = 0.06	ρ = − 0.06, P = 0.69	r = 0.18, P = 0.19
Hyperemic flow velocity	r = − 0.15, P = 0.27	ρ = 0.14, P = 0.63	r = 0.14, P = 0.33
Absolute flow mediated dilation	r = − 0.20, P = 0.14	ρ = − 0.01, P = 0.94	r = − 0.01, P = 0.97
Nitroglycerin mediated dilation	*r = − 0.35 P = 0.03*	ρ = 0.27, P = 0.10	r = − 0.13, P = 0.44

Italic values indicate p < 0.05

**Fig. 2:**
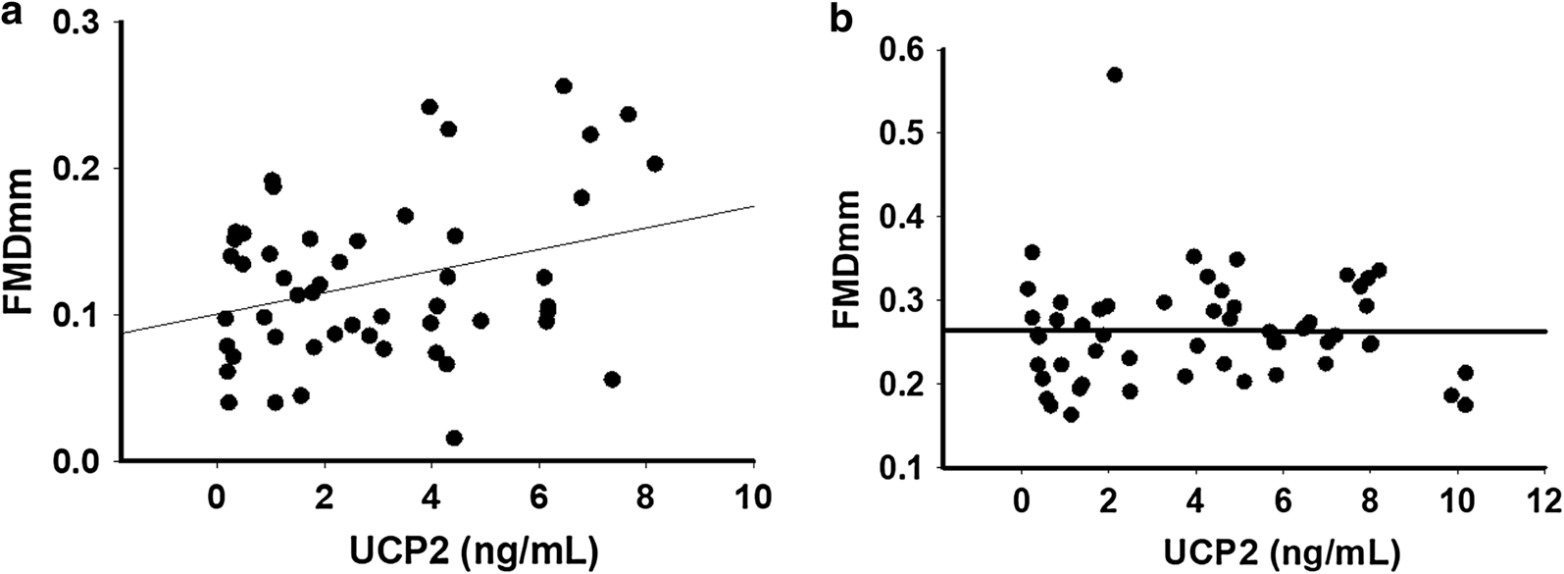
**a**, **b** Flow-mediated dilation (FMDmm) of the brachial artery positively correlated with serum UCP2 levels (r = − 0.30, P = 0.03) (**a**). No correlation between FMDmm and PHB levels were seen (ρ = − 0.052, P = 0.73) (**b**)

In T2DM subjects, prohibitin levels were inversely correlated with resting flow velocity (ρ = − 0.28, p = 0.048) but showed no correlation with FMDmm, NMDmm, resting brachial diameter, or hyperemic flow velocity. UCP2 levels significantly and positively correlated with FMDmm (Fig. 2a, r = 0.30, P = 0.03) but not with any other vascular measurement. Δψ_m_ did not correlate with any of the vascular measurements.

In healthy controls, Prohibitin levels were positively correlated with resting brachial artery diameter (r = 0.28, P = 0.049) but showed no correlation with other vascular measurements. UCP2 levels did not correlate with any vascular measurements. Δψ_m_ significantly and positively correlated with NMDmm (r = 0.35, P = 0.03) but did not correlate with any other vascular measurements.

### Age and sex-adjusted models for measures of endothelial function

In subjects with T2DM, UCP2 remained independently associated with FMDmm in an age- and sex-adjusted models (pooled R^2^ ranging 0.14, B = 0.006 for UCP2; P = 0.049). Prohibitin was no longer independently associated with resting flow velocity in T2DM subjects following adjustment for age and sex (P = 0.15).

In healthy control subjects, age- and sex-adjustment similarly mitigated the association between resting brachial artery diameter and circulating prohibitin levels (P = 0.16). Δψ_m_ remained independently predictive of NMDmm after age- and sex-adjustment (Β = 0.007, P = 0.02).

### Multivariable adjusted models for FMDmm and mitochondrial biomarkers

The results for these multivariable models are included in Table 7 (Control) and Table 8 (T2DM). For healthy controls (Table 7), none of the mitochondrial biomarkers (UCP2, PHB, and Δψ_m_) were independent predictors of FMDmm while both fasting plasma glucose level and sex were independent predictors in all models. For individuals with T2DM (Table 8), UCP2 levels were positive and independently associated with FMDmm following adjustment for age, sex, BMI ≥ 30 kg/m^2^, fasting plasma glucose level, and LDL cholesterol. Of these variable, only SBP was independently (negatively) associated with FMDmm. No variables were independently predictive of FMDmm models with PHB and Δψ_m_,

**Table 7:** Multivariable models predicting FMDmm using Δψ_m_, prohibitin, and UCP2 with clinical variables in healthy control subjects

	Δψ_m_	Prohibitin	UCP2
Age	Β = − 0.0002, P = 0.76	Β = − 0.0005, P = 0.65	Β = − 0.0004, P = 0.69
Sex	*Β = 0.049, P < 0.001*	*Β = 0.047, P < 0.001*	*Β = 0.048, P < 0.001*
SBP ≥ 130 mmHg	Β = 0.015, P = 0.33	Β = 0.016, P = 0.28	Β = 0.017, P = 0.35
BMI ≥ 30 kg/m^2^	Β = 0.011, P = 0.33	Β = 0.008, P = 0.55	Β = 0.011, P = 0.35
Fasting blood glucose	*Β = 0.002, P = 0.001*	*Β = 0.002, P = 0.001*	*Β = 0.002, P = 0.001*
Plasma LDL cholesterol	Β = − 0.0003, P = 0.11	Β < 0.001, P = 0.15	Β = − 0.0002, P = 0.13
Mitochondrial biomarker	Β = 0.00003, P = 0.95	Β = 0.001, P = 0.58	Β = − 0.001, P = 0.82

Italic values indicate p < 0.05

*BMI* body mass index, *SBP* systolic blood pressure

**Table 8:** Multivariable models predicting FMDmm using Δψ_m_, prohibitin, and UCP2 with clinical variables in subject with type 2 diabetes

	Δψ_m_	Prohibitin	UCP2
Age	Β = 0.0003. P = 0.84	Β = − 0.0003, P = 0.77	Β = − 0.0006. P = 0.52
Sex	Β = 0.008, P = 0.71	Β = 0.023, P = 0.19	Β = 0.013, P = 0.44
SBP ≥ 130 mmHg	Β = − 0.02, P = 0.32	Β = − 0.017, P = 0.30	Β = − 0.02, P = 0.21
BMI ≥ 30 kg/m^2^	Β = 0.008, P = 0.33	Β = 0.008, P = 0.72	Β = − 0.004, P = 0.82
Fasting blood glucose	Β = − 0.0001, P = 0.52	Β = − 0.00005, P = 0.81	Β = − 0.00004, P = 0.85
Plasma LDL level	Β = − 0.00006, P = 0.85	Β = − 0.00001, P = 0.98	Β = 0.0001, P = 0.63
Mitochondrial biomarker	Β = 0.0002, P = 0.98	Β = − 0.001, P = 0.60	*Β = 0.007, P = 0.047*

Italic values indicate p < 0.05

*BMI* body mass index, *SBP* systolic blood pressure

## Discussion

In this cross-sectional study, we found population-specific correlation of vascular measurements with measurements of circulating levels of mitochondrial proteins (UCP2 and PHB) involved with modulating Δψ_m_ and mitochondrial superoxide production, as well as Δψ_m_ itself. We found that serum UCP2 concentrations were lower in T2DM subject than controls and positively correlated with FMDmm even following multivariable adjustment. Serum PHB levels weakly correlated with resting brachial diameter (in controls only) and resting flow velocities (in T2DM only). These associations disappeared after adjustment for age and sex. Δψ_m_ remained an independent, positive predictor of NMDmm following age- and sex-adjustment in control subjects only. Additionally, in both T2DM subjects and controls we found and inverse association between UCP2 and PHB levels. To our knowledge, these data are the first to compare circulating levels of UCP2 and PHB in T2DM subjects versus non-T2DM subjects and to correlate these measurements of circulating UCP2 and PHB with measurement of vascular function. Our findings also suggest circulating UCP2 may be a useful biomarker of endothelial health in humans with T2DM- a concept that merits further investigation in larger studies.

UCP2 is the main isoform of uncoupling proteins present in endothelial cells and there is an increasing evidence of animal models that overexpression of UCP2 regulates mitochondrial ROS production and inner mitochondrial membrane potential [21, 35, 36]. UCP2 protects endothelium-dependent vasodilation under hyperglycemic conditions by reducing oxidative stress [37]. In an obese diabetic mice, upregulation of UCP2 improved endothelium dependent vasodilation through increasing endothelium-derived NO and reduced ROS production [38]. Increased vascular expression of UCP2 in the setting of diabetes appears to be an initial compensatory response to systemic elevations in glucose that disappears over time with more chronic and severe abnormal glucose exposures on the diabetic endothelium [39]. Interestingly, a common polymorphism in the promoter of the UCP2 gene (− 866G>A) results in reduced UCP2 expression [40], increased systemic oxidative stress, and an increased risk of myocardial infarction in men with T2DM independent of other traditional risk facts [41]. Our findings extend these data in two ways. First, our data showing a positive association of circulating UCP2 and FMD, but not NMD, provide the first corroborating evidence in humans that UCP2 is associated with endothelial production of nitric oxide. In addition, our data suggest that in individuals with T2DM, circulating levels of UCP2 may reflect overall health of the vascular endothelium and may reflect the residual ability of the endothelium to compensate for excessive oxidative stress and inflammation induced by exposure to elevated glucose levels based on the prior UCP2 work previously delineated. Taken together, our data suggest investigations of serum UCP2 levels as a biomarker of vascular risk in large datasets of T2DM patients together with further work to better delineate the relationship between UCP2 and vascular function are both warranted.

We identified that fasting glucose levels inversely correlated with UCP2 levels in healthy controls but not in T2DM patients. Recent data suggest differences UCP2 expression driven by common polymorphisms in the UCP2 promoter region may play a critical role in insulin secretion and ultimately the development of diabetes [42]. These data suggest that there may be an association between glucose exposure on UCP2 level in healthy individuals and insulin-resistant, non-DM subjects that may be more confounded in individuals with diabetes who also have larger glucose excursions and use therapies to improve glycemic control that modulate insulin secretion, activity, and sensitivity.

In T2DM patients, we also identified circulating UCP2 levels negatively correlating with total cholesterol levels positively correlated with BMI and negatively correlated with in univariate analyses. The cholesterol finding is consistent with animal data showing suppression of UCP2 expression with a high-cholesterol diet [43]. This positive correlation with BMI differs from prior data suggesting UCP2 gene expression in adipose tissue increases with weight loss and that genetic polymorphisms that reduce tissue-level UCP2 expression area associated with increased BMI [44, 45]. The difference in findings might related to circulating UCP2 level not reflecting expression levels in adipocytes or beta cells. Further work is necessary to determine the driving forces behind plasma UCP2 expression in T2DM patients.

PHB is involved cell signaling, aging, regulation of transcription and acts a chaperone for imported proteins in the mitochondria [46–49]. Several studies have shown that over-expression of prohibitin protects cells from oxidative stress injury and its expression in cultured cardiomyocytes and in pancreatic β-cells [46, 50]. PHB expression stabilizes the mitochondrial oxidative phosphorylation system in the inner mitochondrial membrane [47]. In the endothelium, knockdown of the PHB1 isoform results in increased mitochondrial ROS production and reduced angiogenesis [51]. While we did not find an associations between circulating PHB levels and measure of vascular function that survived age- and sex-adjustment, we did find a significant inverse correlation between UCP2 and PHB levels in subjects with T2DM. These data might suggest a compensatory increase in UCP2 expression in the setting of lower PHB levels and vice versa as seen in cultured renal epithelial cells [19]. Increasing UCP2 expression has the effect of lowering Δψ_m_ polarization and mitochondrial superoxide production [7, 52]. Further studies are needed to better delineate the relationships between UCP1 expression, PHB expression, and Δψ_m_ in humans with T2DM.

Δψ_m_ remained an independent, positive predictor of NMDmm following multivariable adjustment in control subjects only. This finding reflects an association between vascular smooth reactivity to nitric oxide in non-DM adults may reflect variations in mitochondrial metabolic activity and merits further investigation [53]. The lack of correlation of Δψ_m_ with FMDmm measurements in either controls or T2DM subjects is entirely consistent with our prior report [7]. The favorable effect of partial depolarization of the mitochondrial inner membrane on resistance artery endothelium-dependent vasodilation to acetylcholine seen in our prior report likely reflects the fact that in T2DM patients basal Δψ_m_ measurements are largely not within a dynamic range that is associated with differences in nitric oxide bioavailability but that treatments to reduce Δψ_m_ polarization reduce mitochondrial superoxide production enough to increase endothelial nitric oxide production.

Our study has some limitations. We measured circulating levels of UCP2 and PHB and Δψ_m_ by isolating circulating mononuclear cells. Levels of circulating markers may not reflect similar levels in the in vivo endothelium. Mitochondrial reactive oxygen species levels corresponding to the UCP2 and PHB levels were not measured in these subjects. The size of the study population (N = 107) is relatively small which limited our ability to develop larger multivariable models. However, our data provide important guidance and support for investing in larger investigations of UCP2 as a biomarker of vascular health and risk in individuals with T2DM as well as studies of how glycemic control might affect UCP2 levels. While fasting glucose levels were available, insulin levels and glycosylated hemoglobin levels will not available for a majority of subjects and therefore we could not correlate these mitochondrial biomarkers with measures of insulin sensitivity and longer-term glucose control. We did have HgA1C levels available for 21 with T2DM, 28 in healthy controls and in this sub-set there were no correlations between UCP2 levels and HgA1C (data not shown). We also did not measure circulating inflammatory markers and so cannot correlate these with UCP2 or PHB. Balanced against these limitations are the novel insights into the association of circulating UCP2 and PHB levels with T2DM and measures of vascular health that support additional studies in large cohorts to further validate these initial findings.

## Conclusion

We found that circulating UCP2 concentrations were inversely correlated with circulating PHB levels and, in T2DM patients, UCP2 concentration were positively associated with conduit vessel endothelium-dependent vasodilation. These data suggest circulating UCP2 concentrations may reflect underlying vascular endothelial function in humans and merits additional investigation as a biomarker of vascular function and potential cardiovascular risk in patients with T2DM. Additional studies looking at how glycemic control in T2DM may be reflected in changes in circulating UCP2 levels are also warranted.

## Data Availability

The datasets used and/or analyzed during the current study are available from the corresponding author on reasonable request

## Abbreviations

Δψ_m_: inner mitochondrial membrane potential
FMDmm: absolute flow-mediated dilation
JC-1: 5,5′,6,6′-tetrachloro-1,1′3,3′-tetraethylbenzamidazol-carboncyanine
NMDmm: absolute nitroglycerin-mediated dilation
PHB: prohibitin
T2DM: type 2 diabetes
UCP2: uncoupling protein 2

## References

[1] International Diabetes Federation. IDF. Diabetes Atlas, 8th edn. 2017. http://www.diabetesatlas.org.

[2] Association AD (2010). Diagnosis and classification of diabetes mellitus. Diabetes Care.

[3] Cersosimo E, DeFronzo RA (2006). Insulin resistance and endothelial dysfunction: the road map to cardiovascular diseases. Diabetes Metab Res Rev..

[4] Baron AD (2002). Insulin resistance and vascular function. J Diabetes Complic.

[5] Zhang DX, Gutterman DD (2007). Mitochondrial reactive oxygen species-mediated signaling in endothelial cells. Am J Physiol Heart Circ Physiol..

[6] Brownlee M (2001). Biochemistry and molecular cell biology of diabetic complications. Nature.

[7] Kizhakekuttu TJ, Wang J, Dharmashankar K, Ying R, Gutterman DD, Vita JA, Widlansky ME (2012). Adverse alterations in mitochondrial function contribute to type 2 diabetes mellitus-related endothelial dysfunction in humans. Arterioscler Thromb Vasc Biol.

[8] Tanner MJ, Wang J, Ying R, Suboc TB, Malik M, Couillard A, Branum A, Puppala V, Widlansky ME (2017). Dynamin-related protein 1 mediates low glucose-induced endothelial dysfunction in human arterioles. Am J Physiol Heart Circ Physiol..

[9] Widlansky ME, Wang J, Shenouda SM, Hagen TM, Smith AR, Kizhakekuttu TJ, Kluge MA, Weihrauch D, Gutterman DD, Vita JA (2010). Altered mitochondrial membrane potential, mass, and morphology in the mononuclear cells of humans with type 2 diabetes. Transl Res..

[10] Shenouda SM, Widlansky ME, Chen K, Xu G, Holbrook M, Tabit CE, Hamburg NM, Frame AA, Caiano TL, Kluge MA, Duess MA, Levit A, Kim B, Hartman ML, Joseph L, Shirihai OS, Vita JA (2011). Altered mitochondrial dynamics contributes to endothelial dysfunction in diabetes mellitus. Circulation.

[11] Li N, Karaca M, Maechler P (2017). Upregulation of UCP2 in beta-cells confers partial protection against both oxidative stress and glucotoxicity. Redox Biol..

[12] La Sala L, Mrakic-Sposta S, Micheloni S, Prattichizzo F, Ceriello A (2018). Glucose-sensing microRNA-21 disrupts ROS homeostasis and impairs antioxidant responses in cellular glucose variability. Cardiovasc Diabetol..

[13] Wang J, Alexanian A, Ying R, Kizhakekuttu TJ, Dharmashankar K, Vasquez-Vivar J, Gutterman DD, Widlansky ME (2012). Acute exposure to low glucose rapidly induces endothelial dysfunction and mitochondrial oxidative stress: role for AMP kinase. Arterioscler Thromb Vasc Biol.

[14] Ren X, Ren L, Wei Q, Shao H, Chen L, Liu N (2017). Advanced glycation end-products decreases expression of endothelial nitric oxide synthase through oxidative stress in human coronary artery endothelial cells. Cardiovasc Diabetol..

[15] Dromparis P, Michelakis ED (2013). Mitochondria in vascular health and disease. Annu Rev Physiol.

[16] Pierelli G, Stanzione R, Forte M, Migliarino S, Perelli M, Volpe M, Rubattu S (2017). Uncoupling protein 2: a key player and a potential therapeutic target in vascular diseases. Oxid Med Cell Longev..

[17] Diano S, Horvath TL (2012). Mitochondrial uncoupling protein 2 (UCP2) in glucose and lipid metabolism. Trends Mol Med..

[18] Marti A, Larrarte E, Novo F, Garcia M, Martinez J (2001). UCP2 muscle gene transfer modifies mitochondrial membrane potential. Int J Obes..

[19] Ye J, Li J, Xia R, Zhou M, Yu L (2015). Prohibitin protects proximal tubule epithelial cells against oxidative injury through mitochondrial pathways. Free Radic Res.

[20] Kang T, Lu W, Xu W, Anderson L, Bacanamwo M, Thompson W, Chen YE, Liu D (2013). MicroRNA-27 (miR-27) targets prohibitin and impairs adipocyte differentiation and mitochondrial function in human adipose-derived stem cells. J Biol Chem.

[21] Lee K-U, Lee IK, Han J, Song D-K, Kim YM, Song HS, Kim HS, Lee WJ, Koh EH, Song K-H (2005). Effects of recombinant adenovirus-mediated uncoupling protein 2 overexpression on endothelial function and apoptosis. Circ Res.

[22] Blanc J, Alves-Guerra M, Esposito B, Rousset S, Gourdy P, Ricquier D, Tedgui A, Miroux B, Mallat Z (2003). Protective role of uncoupling protein 2 in atherosclerosis. Circulation.

[23] Xiong S, Wang P, Ma L, Gao P, Gong L, Li L, Li Q, Sun F, Zhou X, He H, Chen J, Yan Z, Liu D, Zhu Z (2016). Ameliorating endothelial mitochondrial dysfunction restores coronary function via transient receptor potential vanilloid 1-mediated protein kinase a/uncoupling protein 2 pathway. Hypertension.

[24] Widlansky ME, Gokce N, Keaney JF, Vita JA (2003). The clinical implications of endothelial dysfunction. J Am Coll Cardiol.

[25] Thijssen DH, Black MA, Pyke KE, Padilla J, Atkinson G, Harris RA, Parker BA, Widlansky ME, Tschakovsky ME, Green DJ (2010). Assessment of flow mediated dilation (FMD) in humans: a methodological and technical guideline. Am J Physiol Heart Circ Physiol..

[26] Corretti MC, Anderson TJ, Benjamin EJ, Celermajer D, Charbonneau F, Creager MA, Deanfield J, Drexler H, Gerhard-Herman M, Herrington D, Vallance P, Vita J, Vogel R (2002). Guidelines for the ultrasound assessment of endothelial-dependent flow-mediated vasodilation of the brachial artery: a report of the International Brachial Artery Reactivity Task Force. J Am Coll Cardiol.

[27] Suboc TB, Strath SJ, Dharmashankar K, Coulliard A, Miller N, Wang J, Tanner MJ, Widlansky ME (2014). Relative importance of step count, intensity, and duration on physical activity’s impact on vascular structure and function in previously sedentary older adults. J Am Heart Assoc..

[28] Dharmashankar K, Welsh A, Wang J, Kizhakekuttu TJ, Ying R, Gutterman DD, Widlansky ME (2012). Nitric oxide synthase-dependent vasodilation of human subcutaneous arterioles correlates with noninvasive measurements of endothelial function. Am J Hypertens.

[29] Kizhakekuttu TJ, Gutterman DD, Phillips SA, Jurva JW, Arthur EI, Das E, Widlansky ME (2010). Measuring FMD in the brachial artery: how important is QRS-gating?. J Appl Physiol..

[30] Malik M, Suboc TM, Tyagi S, Salzman N, Wang J, Ying R, Tanner MJ, Kakarla M, Baker JE, Widlansky ME (2018). *Lactobacillus plantarum* 299v supplementation improves vascular endothelial function and reduces inflammatory biomarkers in men with stable coronary artery disease. Circ Res.

[31] Widlansky ME, Puppala VK, Suboc TM, Malik M, Branum A, Signorelli K, Wang J, Ying R, Tanner MJ, Tyagi S (2017). Impact of DPP-4 inhibition on acute and chronic endothelial function in humans with type 2 diabetes on background metformin therapy. Vasc Med..

[32] Tang X, Luo YX, Chen HZ, Liu DP (2014). Mitochondria, endothelial cell function, and vascular diseases. Front Physiol..

[33] Nilius B, Droogmans G (2001). Ion channels and their functional role in vascular endothelium. Physiol Rev.

[34] Team RC (2013). R: a language and environment for statistical computing.

[35] Arsenijevic D, Onuma H, Pecqueur C, Raimbault S, Manning BS, Miroux B, Couplan E, Alves-Guerra M-C, Goubern M, Surwit R (2000). Disruption of the uncoupling protein-2 gene in mice reveals a role in immunity and reactive oxygen species production. Nat Genet.

[36] Cabrera JA, Ziemba EA, Colbert R, Kelly RF, Kuskowski M, Arriaga EA, Sluiter W, Duncker DJ, Ward HB, McFalls EO (2012). Uncoupling protein-2 expression and effects on mitochondrial membrane potential and oxidant stress in heart tissue. Transl Res..

[37] Sun J, Pu Y, Wang P, Chen S, Zhao Y, Liu C, Shang Q, Zhu Z, Liu D (2013). TRPV1-mediated UCP2 upregulation ameliorates hyperglycemia-induced endothelial dysfunction. Cardiovasc Diabetol..

[38] Tian XY, Wong WT, Xu A, Lu Y, Zhang Y, Wang L, Cheang WS, Wang Y, Yao X, Huang Y (2012). Uncoupling protein-2 protects endothelial function in diet-induced obese mice. Circ Res.

[39] Cui Y, Xu X, Bi H, Zhu Q, Wu J, Xia X, Qiushi R, Ho PC (2006). Expression modification of uncoupling proteins and MnSOD in retinal endothelial cells and pericytes induced by high glucose: the role of reactive oxygen species in diabetic retinopathy. Exp Eye Res.

[40] Krempler F, Esterbauer H, Weitgasser R, Ebenbichler C, Patsch JR, Miller K, Xie M, Linnemayr V, Oberkofler H, Patsch W (2002). A functional polymorphism in the promoter of UCP2 enhances obesity risk but reduces type 2 diabetes risk in obese middle-aged humans. Diabetes.

[41] Dhamrait SS, Stephens JW, Cooper JA, Acharya J, Mani AR, Moore K, Miller GJ, Humphries SE, Hurel SJ, Montgomery HE (2004). Cardiovascular risk in healthy men and markers of oxidative stress in diabetic men are associated with common variation in the gene for uncoupling protein 2. Eur Heart J.

[42] Gomathi P, Samarth AP, Raj N, Sasikumar S, Murugan PS, Nallaperumal S, Selvam GS (2019). The -866G/A polymorphism in the promoter of the UCP2 gene is associated with risk for type 2 diabetes and with decreased insulin levels. Gene.

[43] Castillo RL, Herrera EA, Gonzalez-Candia A, Reyes-Farias M, de la Jara N, Pena JP, Carrasco-Pozo C (2018). Quercetin prevents diastolic dysfunction induced by a high-cholesterol diet: role of oxidative stress and bioenergetics in hyperglycemic rats. Oxid Med Cell Longev..

[44] Cortes-Oliveira C, Nicoletti CF, de Souza Pinhel MA, de Oliveira BA, Quinhoneiro DC, Noronha NY, Marchini JS, da Silva Junior WA, Junior WS, Nonino CB (2017). UCP2 expression is associated with weight loss after hypocaloric diet intervention. Eur J Clin Nutr.

[45] Brondani LA, Assmann TS, de Souza BM, Boucas AP, Canani LH, Crispim D (2014). Meta-analysis reveals the association of common variants in the uncoupling protein (UCP) 1–3 genes with body mass index variability. PLoS ONE.

[46] Lee JH, Nguyen KH, Mishra S, Nyomba BG (2010). Prohibitin is expressed in pancreatic β-cells and protects against oxidative and proapoptotic effects of ethanol. FEBS J..

[47] Nijtmans LG, de Jong L, Sanz MA, Coates PJ, Berden JA, Back JW, Muijsers AO, van der Spek H, Grivell LA (2000). Prohibitins act as a membrane-bound chaperone for the stabilization of mitochondrial proteins. EMBO J..

[48] Nijtmans L, Sanz MA, Grivell L, Coates P (2002). The mitochondrial PHB complex: roles in mitochondrial respiratory complex assembly, ageing and degenerative disease. Cell Mol Life Sci..

[49] Terashima M, Kim K-M, Adachi T, Nielsen P, Reth M, Köhler G, Lamers M (1994). The IgM antigen receptor of B lymphocytes is associated with prohibitin and a prohibitin-related protein. EMBO J..

[50] Liu X, Ren Z, Zhan R, Wang X, Wang X, Zhang Z, Leng X, Yang Z, Qian L (2009). Prohibitin protects against oxidative stress-induced cell injury in cultured neonatal cardiomyocyte. Cell Stress Chaperones.

[51] Schleicher M, Shepherd BR, Suarez Y, Fernandez-Hernando C, Yu J, Pan Y, Acevedo LM, Shadel GS, Sessa WC (2008). Prohibitin-1 maintains the angiogenic capacity of endothelial cells by regulating mitochondrial function and senescence. J Cell Biol.

[52] Nishikawa T, Edelstein D, Du XL, Yamagishi S, Matsumura T, Kaneda Y, Yorek MA, Beebe D, Oates PJ, Hammes HP, Giardino I, Brownlee M (2000). Normalizing mitochondrial superoxide production blocks three pathways of hyperglycaemic damage. Nature.

[53] Hartman ML, Shirihai OS, Holbrook M, Xu G, Kocherla M, Shah A, Fetterman JL, Kluge MA, Frame AA, Hamburg NM, Vita JA (2014). Relation of mitochondrial oxygen consumption in peripheral blood mononuclear cells to vascular function in type 2 diabetes mellitus. Vasc Med..

